# Systemic and mammary gland disposition of enrofloxacin in healthy sheep following intramammary administration

**DOI:** 10.1186/s12917-015-0406-9

**Published:** 2015-04-09

**Authors:** Cristina López, Juan José García, Matilde Sierra, María José Diez, Claudia Pérez, Ana Maria Sahagún, Nélida Fernández

**Affiliations:** Pharmacology, Department of Biomedical Sciences, Institute of Biomedicine (IBIOMED), University of León, Campus de Vegazana s/n, 24071 León, Spain; Department of Animal Health, University of León, Campus de Vegazana s/n, 24071 León, Spain

**Keywords:** Enrofloxacin, Sheep, Intramammary, Systemic, Glandular tissue, Disposition

## Abstract

**Background:**

Mastitis is one of the most important diseases affecting dairy sheep. Antimicrobial drugs are often administered directly through teat to treat or prevent this disease, but data on drug distribution within glandular tissue are scarce and it cannot be estimated from concentrations in milk. Thus, the aim of this study was to investigate systemic and mammary gland distribution of enrofloxacin after intramammary administration. The drug was administered to 6 healthy lactating Assaf sheep with an injector containing an enrofloxacin preparation (1 g drug/5 g ointment). Blood samples were collected at 0, 30, 60, 90, 120, 150 and 180 min. Animals were then sedated and sacrificed, and glandular tissue samples were obtained from treated udders at 2, 4, 6 and 8 cm height. Enrofloxacin concentrations were measured in plasma and tissue samples by UV high-performed liquid chromatography.

**Results:**

Mean enrofloxacin plasma concentrations were below 0.5 μg/mL. Mean tissue concentrations decreased in mammary gland with vertical distance from the teat, ranging from 356.6 μg/g at 2 cm to 95.60 μg/g at the base of the udder. Glandular tissue concentrations best fitted to a decreasing monoexponential model, and showed a good correlation with an ex vivo model previously developed.

**Conclusions:**

Enrofloxacin concentrations were effective in the entire glandular tissue against the main pathogens causing mastitis in sheep. These results suggest that this drug may be suitable to treat mastitis in sheep by intramammary administration.

## Background

Mastitis is one of the most important and costly diseases affecting dairy sheep, resulting in substantial health and economic problems worldwide. The two major forms of this disease are clinical mastitis, which results in signs of inflammation in infected mammary glands, changes in milk composition and altered systemic condition of the animal, and subclinical mastitis, without readily apparent signs in animals but with altered somatic cell count of the milk. From both types of mastitis, the subclinical one is not only very frequent but also economically important as it reduces the quantity and quality of milk produced. Prevalence values of this form average 5-30% in small ruminants, whereas for clinical mastitis they are generally lower than 5% [1-3].

Although a variety of microorganisms can cause mastitis in sheep, *Staphylococcus* spp. are the most frequently isolated agents involved in both acute clinical and subclinical forms. Other pathogens such as *Enterobacteriaceae*, *Streptococcus* spp., *Mycoplasma* spp., *Mannheimia haemolytica* or *Pseudomonas* spp. are also found.

Fluoroquinolones are considered among the most effective drugs for the treatment of bacterial infections, and they should be reserved to those situations with poor response to other antimicrobial agents, in order to prevent increasing the risk of quinolone-resistant bacteria. Although this pharmacological group should not be used as first-line agents against mastitis, it is important to establish its pharmacokinetics and efficacy in animals in which it could be employed. Enrofloxacin, 1-cyclopropyl-7-(4-ethyl-1-piperazinyl)-6-fluoro-1,4-dihidro-4-oxo-3-quinoline carboxylic acid, is a fluoroquinolone exclusively developed to be used in veterinary medicine [4,5]. It is characterized by a low host toxicity, a high bioavailability, an excellent tissue penetration, a long serum half-life and a broad antibacterial spectrum together with a high bactericidal activity against major pathogenic bacteria (both Gram-positive and Gram-negative) and intracellular microorganisms found in sick animals [6,7].

The pharmacokinetics of enrofloxacin have been widely described in several species including cattle [8-11], buffaloes [12,13], sheep [14-16] and goats [5,17,18]. In most of these species enrofloxacin shows good absorption after parenteral administration, although oral absorption drops to approximately 10% in adult ruminants [19]. This drug exhibits a large volume of distribution, suggesting wide tissue penetration, and a terminal half-life of 2–6 h. Furthermore, enrofloxacin is partially metabolized in the liver to ciprofloxacin, a primary metabolite which is a potent antimicrobial agent itself [8].

It is well known that the strategies to control mastitis include teat dip disinfection, milking procedures and selective dry-off therapy, but studies on the effectiveness of intramammary antimicrobial drugs are especially important, as many antimicrobial drugs are often administered directly through the streak canal to treat or prevent clinical or subclinical mastitis in dairy sheep. Regarding intramammary administration, to establish rational therapeutic dosage regimens, it is necessary to determine drug concentrations achieved within the udder and if these concentrations are sufficient to kill or inhibit microorganism growth, taking into account that drug distribution in tissue udder can vary according to its liposolubility, its affinity for the tissue and the formulation or the vehicle used. Milk and blood sampling are often employed in vivo to assess local concentrations for practical, ethical and economic reasons, although drug concentrations at different areas of the udder cannot be estimated in this way. In a previous study [20] we have developed an ex vivo model of isolated and perfused udder in sheep to investigate the pharmacokinetic behavior of enrofloxacin. In order to complete this former one, the objective of the present study was to determine plasma and tissue mammary gland concentrations of enrofloxacin in healthy sheep after single intramammary administration of 1 g enrofloxacin (1 g drug/5 g ointment) to evaluate, on one hand, the degree of distribution of enrofloxacin in the mammary gland and, on the other one, the transfer of this drug to systemic circulation. Moreover, the study will allow us to confirm the validity of the isolated and perfused model previously carried out in sheep.

## Methods

### Animals

The study was carried out in six healthy female lactating Spanish Assaf sheep weighing 45–57 kg and aged 4–5 years. Sheep were acclimatized and fed with an antibacterial-free diet of alfalfa hay and pelleted feed concentrate, and unlimited access to water and saltlick. The Institutional Animal Care and Use Committee of the University of León approved in advance all animal procedures described here.

Clinical signs of mastitis, changes in milk or skin lesions were not observed in any gland. Sheep were completely milked previously to drug administration. Intramammary administration was made through teat with a single injector containing an enrofloxacin suspension (1 g of enrofloxacin/5 g of ointment) via the teat canal, massaging afterwards into the gland cistern. Ointment was administered only in one gland. Enrofloxacin preparation was developed by Syva S.A. Laboratories (Leon, Spain), and it is still at the research stage. Up to date, and to our knowledge, there is no enrofloxacin medicine for intramammary use.

Blood samples (10 mL) were collected into heparinized vacuum tubes from a catheter laid in the jugular vein just prior to drug administration, and at 30, 60, 90, 120, 150 and 180 min. Plasma was immediately separated by centrifugation (1500 rpm for 20 min).

After 180 min sampling time, sheep were slaughtered with the following protocol: sedation was carried out with propofol (Propovet (10 mg/mL), Esteve, Barcelona, Spain) (3 mg/kg, intravenous (IV) route) prior to administration of the euthanasia medicine containing embutramide, mebezonium iodide and tetracaine hydrocloride (T-61^©^, Merck Sharp & Dohme Animal Health, Salamanca, Spain) (100 mg/kg, IV route). Samples of glandular tissue were obtained at four different distances (2, 4, 6 and 8 cm) from the base of the teat. All plasma and tissue samples were frozen at −20°C and stored at −80°C until analysis.

### Analysis of enrofloxacin concentrations

Enrofloxacin concentrations in plasma and glandular tissue samples were measured by reversed phase high performance liquid chromatography (HPLC) with UV detection (LC Module I Plus, Waters Corporation, Mildford, MA, USA). 1 mL plasma sample was deproteinized with 0.5 mL 10% acetic acid, centrifuging at 3000 rpm for 10 min. Samples were then extracted and purified according to a method previously reported [21] with minor modifications. Mammary tissue samples were also extracted and purified using a method previously described [22] with minor modifications. 4 mL dichlorometane were added to 1 g tissue and homogenized at 13500 rpm for 30 seconds (Ultra-Turrax T-25, IKA Works Inc, Wilmington, OH). 4 mL dichlorometane and 0.5 mL sodium phosphate buffer 0.5 M (pH = 7.5) were then added to homogenate, which was shaken and centrifuged at 2500 rpm for 10 min. The organic phase was collected and mixed with 1 mL NaOH 0.5 M, centrifuging again at 2500 rpm for 10 min. This latter step was repeated twice, collecting the aqueous phase, which was injected into the chromatograph.

Chromatographic analysis was carried out with a separation Nova Pak C_18_ column (4 μm, 250 × 4 mm) (Waters Corporation, Mildford, MA, USA) at room temperature. The mobile phase consisted of a mixture of a solution of sodium acetate (pH 4.7; 0.1 M) and acetonitrile 60:40 (v/v), with pH adjusted to 5 by addition of acetic glacial acid. The flow rate of the mobile phase was 1 mL/min, and the wavelength was set at 278 nm. Under these conditions, the retention time of enrofloxacin was 2.68 min. Recovery was 73.5 ± 9.1% and 83.8 ± 7.9% in tissue and plasma samples, respectively. Interday and intraday accuracy and precision were within 10%. The limit of quantification was 0.08 μg/mL in both plasma and tissue and the limit of detection was 0.04 μg/mL in plasma and 0.08 μg/mL in tissue.

### Statistical evaluation

Data were reported as mean ± standard deviation (mean ± SD) or median and quartiles. Normality of the data and uniformity of the variance were determined by asymmetry and Levene’s test, respectively. When data were normally distributed two-way ANOVA and Duncan test were used to evaluate the significance of differences in drug concentrations of treated udders. If data were not normally distributed, Friedman and Wilcoxon tests were then employed. A value of *P* ≤ 0.05 was considered as level of significance for all analyses.

## Results

**Figure 1:**
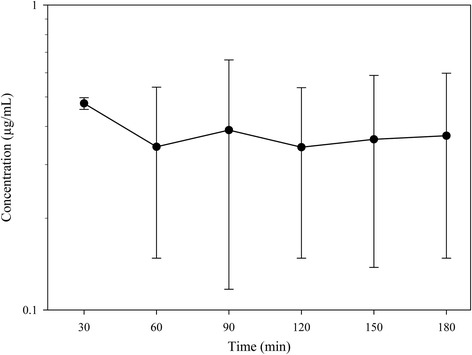
**Plasma enrofloxacin concentrations after intramammary administration to 6 sheep.** Administration was made with an injector containing 1 g enrofloxacin/5 g ointment. Data are given as mean ± SD (semilogarithmic scale).

Mean enrofloxacin plasma concentrations after intramammary administration are shown in Figure 1. Concentrations measured were low in these first 3 h after administration, remaining nearly constant from 60 min onwards. At 180 min concentrations ranged from 0.077 to 0.569 μg/mL. In one of the animals the drug was not detected at any sampling time, and at 30 min enrofloxacin was detected only in two sheep.

**Figure 2:**
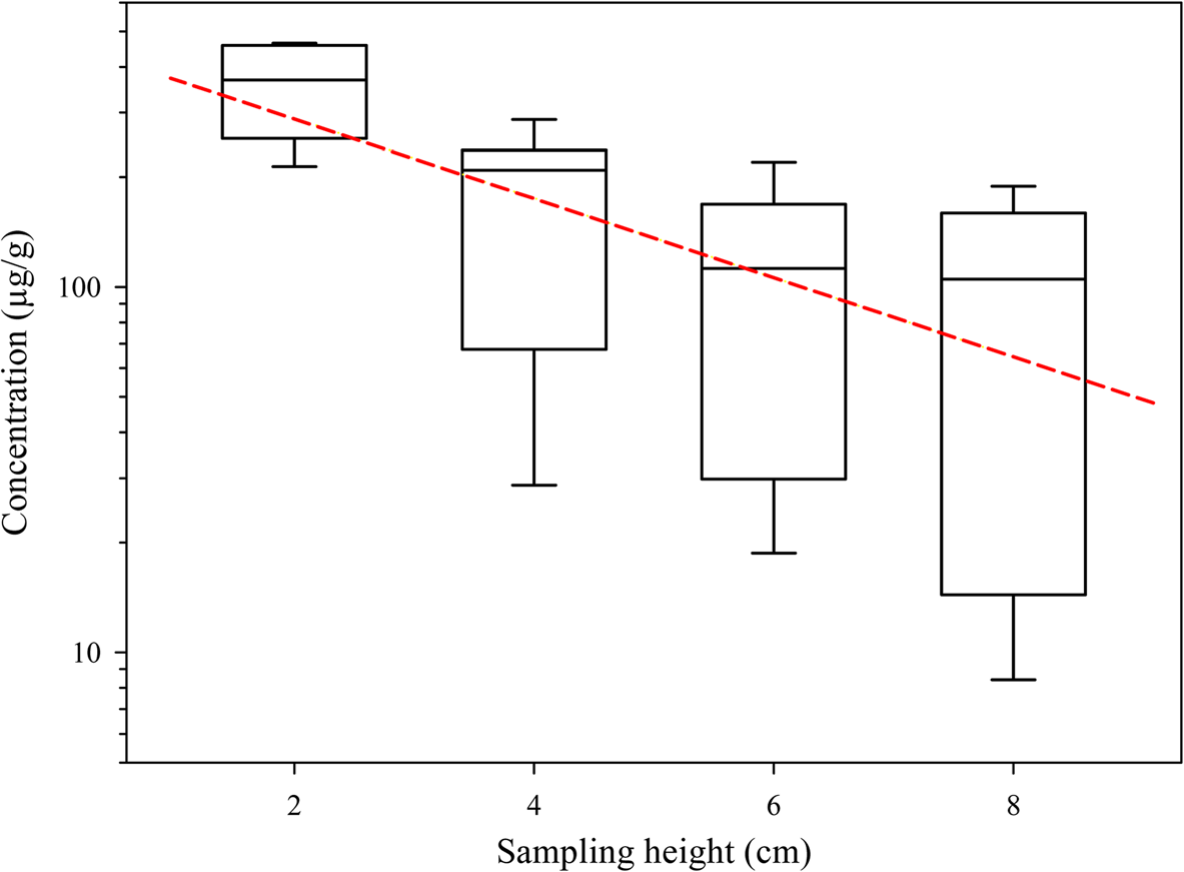
**Enrofloxacin concentrations in glandular tissue after intramammary administration to 6 sheep.** Administration was made with an injector containing 1 g enrofloxacin/5 g ointment. Samples were taken at constant vertical distances from the base of the teat. Significant differences were found between concentrations determined at 2 cm and at the rest of the heights. Theoretical values predicted with the equation Concentration = 478.804 · e^-0.251 · height^ are also included (short dash line). Data are given as box plots with median and quartiles (semilogarithmic scale).

Regarding tissue concentrations, Figure 2 summarizes enrofloxacin concentrations in glandular tissue taken at 180 min at the different sampling heights. Animals were evaluated hourly during the assay, and neither general signs nor local irritation reactions (swelling or tissue hardening) were observed. Drug concentrations always decreased with increasing distance from the teat. Enrofloxacin concentrations were 356.6 ± 109.3 μg/g tissue near the teat (2 cm distance), declining to 172.34 ± 97.3; 113.06 ± 81.8 and 95.60 ± 74.0 μg/g at 4; 6 and 8 cm distances, respectively. At this latter point, at the base of the udder, values ranged from 8.42 to 188.84 μg/g. Significant differences were found among enrofloxacin concentrations determined at 2 cm and those measured at the other heights (Duncan test, *P* ≤ 0.05).

Tissue concentrations obtained in healthy animals were also compared with those determined in the ex vivo isolated and perfused udders after intramammary administration [20], and modelled. In both, healthy sheep and isolated udders, the best fit was obtained with a decreasing monoexponential model, described by the following equations:

$$ \mathrm{Ex}\ \mathrm{vivo}\ \mathrm{model}:\ \mathrm{Concentration} = 177.776 \cdot p {\mathrm{e}}^{\hbox{-} 0.251 \cdot p\ \mathrm{height}}\left({\mathrm{r}}^2 = 0.924;\ \mathrm{p} = 0.039\right) $$

$$ \mathrm{Healthy}\ \mathrm{sheep}:\ \mathrm{Concentration} = 478.804 \cdot p\ {\mathrm{e}}^{\hbox{-} 0.219 \cdot p\ \mathrm{height}}\left({\mathrm{r}}^2 = 0.950;\ \mathrm{p} = 0.025\right) $$

In both equations the slopes are very similar (0.251 for the ex vivo model and 0.219 for healthy sheep), which would reveal a similar diffusion kinetics. Thus, taking into account the coefficients obtained in both equations, it would be possible to predict concentrations in alive animals at different heights of mammary gland by multiplying ex vivo tissue concentrations by 2.693 or by using a new equation obtained from that used to describe the ex vivo model concentrations, multiplying the intercept (177.776) by 2.7:

$$ \mathrm{Concentration} = 478.804 \cdot p\ {\mathrm{e}}^{\hbox{-} 0.251 \cdot p\ \mathrm{height}} $$

Figure 2 shows the close correspondence among the theoretical enrofloxacin concentrations in mammary gland predicted with this latter equation and the experimental values determined in the same tissue but in healthy animals.

## Discussion

As expected, enrofloxacin plasma concentrations were low after intramammary administration, showing that most drug remains in the udder carrying out its bactericidal action. These low concentrations would minimize the risk for consumers due to residues in meat or in other tissues. The drug was detected after 30 min administration only in two animals, which would indicate that in the other sheep the rate of absorption was slower that in these two animals. In one sheep absorption was too small to determine detectable levels in plasma at any sampling time. On the other hand, plasma drug concentrations determined in healthy animals were always lower than those obtained in Tyrode solution with the ex vivo model of isolated and perfused sheep udder [20]. This may be due to the fact that the ex vivo model exhibits a lower volume of distribution and it does not reflect the actual physiological conditions of drug elimination, as it lacks the organs involved in elimination process such as liver or kidneys. On the other hand, in healthy animals, although a greater transfer of drug from mammary gland into the bloodstream is expected in comparison with the ex vivo model, the proportional increase in the elimination process should explain the lower enrofloxacin plasma levels.

Regarding enrofloxacin concentrations in glandular tissue, they decreased progressive and exponentially with increased vertical distance from the base of the teat. The high variability obtained in tissue concentrations is consistent with those results indicated in other similar studies [23-27], and it can be due to interindividual variations related to gland size or milk secretion volume (related to the degree of drug dilution), even though animals were selected according to similar conformation and udder size, level of milk production or days in milk. Enrofloxacin concentrations determined reflect a good distribution of this drug in the entire mammary gland. We have chosen four locations per quarter as they represent the minimum number of samples to assess drug distribution because antimicrobials distribute unevenly in glandular tissue [23,28].

Comparing with drug concentrations determined in the ex vivo model developed in sheep [20], mammary concentrations in healthy animals are higher than those quantified in the ex vivo model. Again, these differences could be due to disparities between the model and the physiological conditions of healthy animals: composition of Tyrode solution is not exactly similar to blood composition, and there could also be differences between perfusion flow in the ex vivo model and blood flow in alive animals. Moreover, the ex vivo model is static whereas alive sheep were allowed to move during the entire duration of the sampling time. Finally, the formulation used can also have an influence on the distribution of antimicrobials administered locally into the bovine udder. All these factors could contribute to a greater diffusion of drug within the mammary gland in alive sheep.

Data on drug distribution in mammary gland are scarce, and drug concentration in milk after intramammary administration cannot be used to estimate drug concentration in udder tissue [25]. At present two different approaches have been followed. On one hand, ex vivo perfused models have been designed to establish drug concentrations in bovine and ovine udders [20,24,25,29] but it remained unclear if they accurately reflect the situation in vivo [26,30]. On the other one, anesthetized alive animals have been used to determine tissue distribution, but its major inconvenient is that quarters can be sampled only once [31], which actually limits its usage. We have chosen an intermediate approach to evaluate if drug concentrations determined in healthy animals are comparable to those obtained in the ex vivo perfused model [20]. In this case, we have fixed 180 min as sampling time to collect tissue samples as it is the time for which the isolated and perfused udders remained viable in sheep.

Finally, to establish if sufficient antibacterial concentrations are reached in the target tissue after enrofloxacin intramammary administration, a pharmacokinetic/pharmacodynamic (PK/PD) evaluation was also developed to assess treatment effectivity. Regarding its antibacterial activity, enrofloxacin, as any fluoroquinolone, has a concentration-dependent activity. Minimal inhibitory concentrations that inhibits 90% of bacterial isolates (MIC_90_) have been categorized as sensitive (<0.25 μg/mL), intermediate (0.5-1 μg/mL) or resistant (>2 μg/mL) [32]. In sheep bacterial isolates a MIC of 0.5 μg/mL has been established for *Mycoplasma agalactiae* [33]. MIC values determined in cattle isolates are 0.12 μg/mL for *Escherichia coli* [34], 0.125 μg/mL for *Mannheimia haemolytica* [35], and 0.25 μg/mL for *Staphylococcus aureus* [36-38] and *Streptococcus* spp. [39]. In all the animals MIC were exceeded in the entire glandular tissue following intramammary administration, whereas in plasma samples mean enrofloxacin concentrations did not achieve the MIC fixed for *Mycoplasma agalactiae*.

Moreover, we have chosen the PK/PD index Concentration/MIC (C/MIC) > 8–10 [40,41] to assess if sufficient antibacterial concentrations are reached after intramammary administration within the glandular tissue. After 3 h drug diffusion, enrofloxacin concentrations in the entire glandular tissue were always more than 8–10 times above the MIC established for the main pathogens causing mastitis in sheep. In the base of the udder, C/MIC was 191.20 for *M. agalactiae*; 382.40 for *S. aureus* and *Streptococcus* spp.; 764.80 for *M. haemolytica* and 796.67 for *E. coli*. Regarding plasma concentrations, intramammary treatment would not be actually effective against any microorganism, as C/MIC was always lower than 8.

Finally, and with respect to the potential presence of enrofloxacin residues in both tissues or milk after intramammary administration, the study has shown that the transference of the drug from the mammary gland to plasma is low, which would diminish the risk of drug residues in meat. In mammary gland tissue, high enrofloxacin concentrations have been determined. Thus, further studies are necessary to ensure that the drug has been completely excreted from the animal, prior to dairy/meat products being available for consumers, in order to avoid the emergence and dissemination of harmful bacteria resistant to fluoroquinolones and/or undesirable effects on consumers.

## Conclusions

Fluoroquinolones should be only used to treat serious infections with bacteria resistant to other antibacterial agents. To our knowledge, this is the first time that tissue distribution of enrofloxacin in the mammary gland and its degree of systemic absorption following intramammary administration have been established. The study provides complementary data on enrofloxacin tissue distribution that could be useful to design formulations employed to treat mastitis in this species. The drug shows a low transfer to systemic circulation and a wide distribution throughout the udder, achieving in the entire gland adequate concentrations against the major mastitis pathogens in sheep. Thus, enrofloxacin may be a suitable option for treating this disease in dairy sheep following intramammary administration although further studies are needed to ensure its efficacy and safety.

## Abbreviations

IV: Intravenous
HPLC: High performance liquid chromatography
MIC_90_: Minimal inhibitory concentration that inhibits 90% of bacterial isolates
PK/PD: Pharmacokinetic/pharmacodynamic
C/MIC: Concentration/MIC
